# The mechanism of Tat-dependent protein translocation

**DOI:** 10.15698/mic2026.05.875

**Published:** 2026-05-15

**Authors:** Thomas Brüser, Carsten Sanders

**Affiliations:** 1 Institute of Microbiology, Leibniz University Hannover, 30419 Hannover, Germany; 2 Department of Biology, Millersville University, Millersville, PA 17551, USA; 3 Department of Biology and Integrative Science, School of Science, Engineering and Technology, Penn State Harrisburg, Middletown, PA 17057, USA; 4 Chemistry Division, Penn State York, York, PA 17403, USA

**Keywords:** twin-arginine translocation, protein transport, membrane proteins, protein interactions, biological membranes, transport energetization

## Abstract

The twin-arginine translocation (Tat) system is the only general pathway for the transport of folded proteins across energized biological membranes. It is found in the bacterial or archaeal cytoplasmic membrane, the plant thylakoid membrane or the inner membrane of plant mitochondria. The biological importance of this translocation system can be exemplified by the fact that all bacterial or plant photosynthesis and photosynthetic oxygen evolution on earth requires this system. Despite many biochemical and biophysical studies, the Tat mechanism has been puzzling since the system was discovered in the 1990ies. Important characteristics of the Tat system could not be explained, and also recent high-resolution structures of the Tat system’s core with bound substrate has not led to a general transport mechanism yet. In this integrative review, we attempted to answer the key open questions relevant to the Tat mechanism and thereby developed an in its molecular detail new comprehensive explanation of how folded proteins are translocated across membranes by the Tat system.

## INTRODUCTION

**Figure 1:**
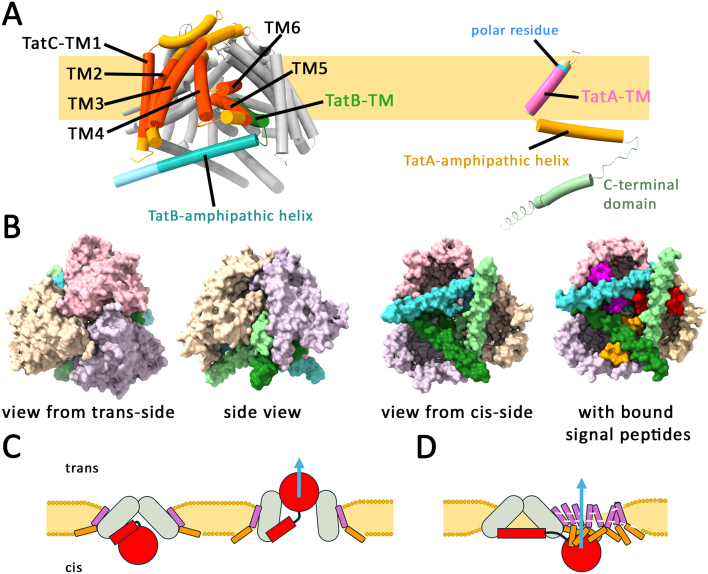
The components of the Tat pathway and the contradictory initial interpretations of the TatBC complex structure. **(A)** The topology of TatB and TatC in the TatBC complex, and the topology of a single TatA in the membrane. The positions of the transmembrane helices of TatC (TM1-6) as well as the positions of the transmembrane helices and amphipathic helices of TatB and TatA are indicated. Note that usually a polar residue (blue) limits the stretch of hydrophobic residues in the TM of TatA to only 12 residues, which is important for membrane weakening. In some cases, such as *B. subtilis* TatA ${}_{d}$ , a helix-breaking PG motif is found instead of the polar residue, which can serve the same function. TatB is structurally related to TatA, but its TM is in an unusual orientation and positioned in the center of the resting TatBC complex. The structure of TatBC is taken from Deme et al. (PDB 9DZZ, Ref. 9, and the structure of TatA has been generated by AF3 15. **(B)** Views on the TatB ${}_{3}$ C ${}_{3}$ complex from indicated perspectives, in one case with fully occupied primary signal peptide binding sites (based on PDB 9DZZ and 9E01). The three TatC subunits are in light beige, light pink and light lilac. The three TatB subunits are in three shades of green/turquoise. **(C)** and **(D)**: Schematic cross sections through transporting Tat systems, highlighting the different substrate passages proposed by Zhao and Sazanov (**C**, 8) and Deme et al. (**D**, 9), who independently solved the TatBC complex structure with bound substrate. Color code: gray, TatC; pink/orange, TatA transmembrane helix and amphipathic helix; red, substrate.

Tat-dependently translocated proteins have N-terminal signal peptides with a highly conserved twin-arginine (RR) motif that is recognized by a TatBC complex in the membrane before a TatA-dependent translocation takes place 1. While TatC is a polytopic membrane protein with six transmembrane helices, TatA and TatB have only a short N-terminal transmembrane helix (Figure 1**A**). They belong to the same protein family and require their membrane anchor, a subsequent amphipathic helix and only few more residues for functionality, whereas their not conserved highly hydrophilic C-terminal domain is dispensable 2–4. In three-component systems, one TatA/B family protein, TatB, evolved to tightly interact with TatC to form TatBC complexes that can recognize twin-arginine signal peptides of Tat-dependently translocated proteins, whereas another TatA/B family protein, TatA, enables the membrane passage 1. There also exist two-component TatAC systems in which a “bifunctional” TatA fulfills TatA and TatB functions 5–7. Tat systems are found in all domains of life, particularly in organisms that need a general transport pathway for folded proteins 2. Despite of decades of intensive research, the translocation mechanism was still puzzling and structural insights were needed. In their recent high-resolution Cryo-EM structures of TatBC complexes with bound substrate, the research groups of Sazanov as well as of Berks and Lea revealed many aspects of subunit interactions and substrate recognition on a molecular level 8, 9. The structures show that the TatBC complex is composed of three TatC and three TatB subunits (one of which may be substituted by TatA), which together form a structure resembling an inverted cup with its opening towards the cytoplasm covered by a triangle of amphipathic TatB helices (Figure 1**B**). A signal peptide is bound by interactions with the N-terminus and first cytoplasmic loop of TatC and sandwiched between the amphipathic helix and the N-terminal helix of two TatB protomers, which fully agrees with earlier biochemical and molecular biological data 10–14. However, contradictory translocation modes are proposed (Figure 1**C**): One study favors a channel model with transport through a transient and symmetric opening at the top of the complex, which could further open and extend to a larger size by the aid of TatA in case of larger Tat substrates 8. The other study excludes such an opening and instead favors a lateral passage of Tat substrates through an associated, membrane-thinning TatA oligomer that is recruited by “semi-specific interactions with the folded passenger domain”. The translocation mechanism is hypothesized to be accomplished not by a TatB ${}_{3}$ C ${}_{3}$ but by a structurally asymmetric TatA ${}_{1}$ TatB ${}_{2}$ TatC ${}_{3}$ complex 9. Mechanistic details beyond substrate-binding are left unclear for both proposed translocation modes.

We provide here a conclusive multistep model of how the Tat system transports a folded protein with an appropriate signal peptide across a biological membrane. This model can explain hitherto puzzling facts of the Tat system at a structural level, which is why we believe that it likely represents a major advancement in the molecular understanding of the Tat mechanism. In a first phase (phase I), the signal peptide of a Tat substrate is membrane-inserted by TatB ${}_{3}$ C ${}_{3}$ . In a second phase (phase II), an associated TatA ring destabilizes and disrupts the membrane at the translocation site, thereby permitting translocation. In a third phase (phase III), the system resets and releases the translocated substrate. The system requires membrane energetization for the first two phases and likely a transient local depolarization for resetting during the third phase.

### Tat transport cannot occur through a central pore generated by a TatB_3_C_3_ complex

Zhao and Sazanow hypothesize that the TatB ${}_{3}$ C ${}_{3}$ complex is designed for opening in its center, as only interactions of short parallel beta strands and few other interactions stabilize the proposed closed state of the complex (Figure 1**C**). The complex has highly tilted transmembrane helices in a thinned membrane environment and is therefore very unusual 8. Although the extremely unusual shape of the complex can be expected to be related to the mechanism, Tat substrates cannot pass through the center of a TatBC pore with TatA remaining on the outside, even if it is assumed that a central pore can be laterally extended for larger substrates by the aid of associated TatA. There are multiple reasons for this:

(i) If larger TatA assemblies would be required for an optional pore extension only, the signal peptide alone would not cause the same TatA association as observed with full-length Tat substrates, but it does 17. (ii) If TatBC-containing complexes would generally open with a central pore, there would not exist TatBC complexes with interacting large periplasmic loops that prevent opening, such as found in *Myxococcus xanthus* 9. (iii) If transport would proceed through a central TatBC pore, the signal peptide would need to be reinserted into the membrane in order to be cleaved by a signal peptidase (such as LepB), and there is no such pathway known 18. (iv) An efficient but unknown membrane-reinsertion pathway would also be required for Tat substrates that have membrane-integral N- or C-termini and are often part of larger membrane-protein complexes, such as the cytochrome *bc* ${}_{1}$ complex 19, 20. (v) Some Tat substrates are small globular C-terminal domains of membrane proteins whose N-terminal domains are already membrane-inserted when Tat transport of the C-terminal domain takes place. Such a transport would be impossible through a central pore formed by TatB and TatC components 21.

In contrast to Zhao and Sazanow, Deme et al. favor a passage through a laterally associated TatA oligomer that weakens the membrane 9 (Figure 1**D**). Such a translocation step was first suggested by us in 2003 22 and later supported by various experimental data 23–25 and molecular dynamics simulations 26, 27. Based on their analyses, Deme et al. reasoned that one TatA is always in one of the three TatB binding sites under physiological conditions, resulting in a TatA ${}_{1}$ TatB ${}_{2}$ TatC ${}_{3}$ complex 9. They believe that this structural asymmetry in the resting state is functionally needed for activity, and that only one signal peptide binding site is functional in Tat complexes, as only one site is not influenced by a bound TatA. Furthermore, they inferred that the translocation process is initiated after recruitment of a laterally associating TatA assembly by the aid of the globular domain of bound Tat substrates. Mechanistic explanations for a directional translocation through the lateral TatA assembly are not described. However, as outlined below in detail, an intrinsic structural asymmetry of the Tat complex is not mechanistically required and all binding sites can be equally occupied, albeit they probably initiate transport sequentially.

### All substrate binding sites can be equally functional

Based on double-tag purifications and structure analyses, Deme et al. concluded that all core complexes have a TatA, and they suggest that this intrinsic asymmetry is essential 9. They thus question the widely accepted 1:1 TatBC ratio, which relied mainly on slightly variable but convincing quantifications of radioactivity in SDS-PAGE bands of purified labeled Tat complexes 28. Indeed, TatA is associated with TatBC even in the absence of bound Tat substrates 29, and TatA can still be cross-linked to TatC when the system is in the resting state 30, which appears to agree with a TatA ${}_{1}$ TatB ${}_{2}$ TatC ${}_{3}$ complex as proposed by Deme et al. 9. However, various biochemical studies strongly support a TatB ${}_{3}$ TatC ${}_{3}$ composition of the core complex: (i) An increase of TatA-TatC cross-links was observed at TatC transmembrane helix 5 (TM5) in response to active transport, indicating that this TatA interaction is transient and thus likely resulting from a translocation-dependent approximation of TatC and TatA, rather than from a permanent exchange 31. (ii) Pull-down experiments with *E. coli* TatABC preparations showed that TatA-antibodies pulled down only a small fraction of TatBC in comparison to the pull-down with TatB antibodies, with a 1:1 TatB/TatC ratio being retained, indicating that TatA is more loosely bound at other positions. Similarly, when analyzed by BN-PAGE, co-purified TatA readily dissociated, and it has yet been impossible to identify TatA as an integral constituent of the TatBC-containing core complex 28, 32. (iii) The research group of Colin Robinson revealed that an *E. coli* TatA F39A variant strongly altered the TatA interaction with TatBC, as antibody-independently detected by radiolabeled proteins, resulting in a drastically increased TatB/C ratio and absence of interacting TatA in fractions of purified complexes. This showed that the constitutive TatC interaction differs between TatA and TatB. TatA interacts weaker and this interaction depends on positions in the amphipathic helix, and alterations in the TatA association can generate additional binding sites for TatB 33. (iv) Biochemical analyses by the research group of Ken Cline clearly demonstrated that, in the plant Tat system, each individual TatC can bind a substrate, and that all binding sites are independent 34, and finally (v) there are active TatAC systems in *Bacillus* and other genera that simply cannot have this structural asymmetry, as their “bifunctional” TatA takes the roles of TatA and TatB, and it is highly unlikely that these systems are mechanistically significantly different 6. The argument of Deme et al. that overproduced TatB suppresses Tat transport 9 does not show a requirement of a constitutive TatA subunit, as the effect may simply result from the a suppression of efficient TatA cluster association or the formation of non-functional mixed TatAB assemblies. In conclusion, in the resting TatABC-containing system of the *E. coli*-type, TatA is more loosely bound at a different position than TatB, and thus TatB ${}_{3}$ TatC ${}_{3}$ likely forms a stable core complex in which all three signal peptide binding sites are functional, as they are in TatAC complexes of the *Bacillus*-type.

## PHASE I OF TAT TRANSPORT: SIGNAL PEPTIDE INSERTION BY TATBC COMPLEXES

### Previously unanswered key questions that helped to uncover the TatBC complex mechanism:

- How can TatC catalyze the insertion of signal peptides without transport of the mature domain, and why does TatB prevent this insertase activity, as observed by Fröbel et al. 35?
- If this insertase activity is important for the mechanism, how can the signal peptide insertion be catalyzed without liberating the twin-arginine motif from its initial binding site, as observed by Gerard et al. 36?
- How can signal peptide binding to TatBC complexes alone already cause the association of TatA clusters, as observed by Dabney-Smith et al. 17?

### TatB N-terminal helices block access to the signal peptide h-region binding site in TatC

**Figure 2:**
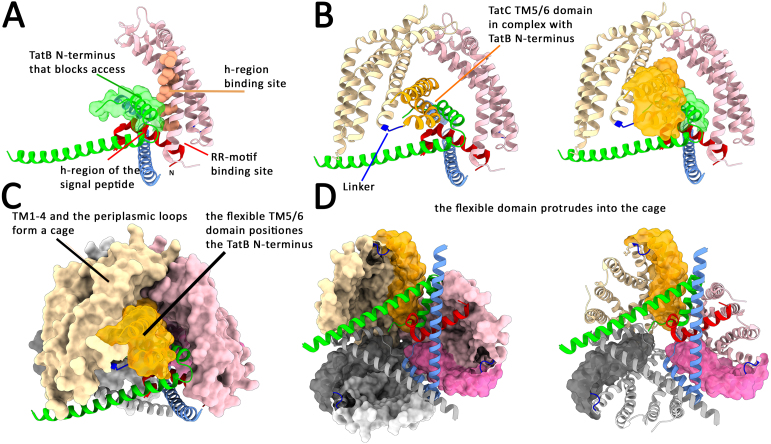
The TatC TM5/TM6 domain positions TatB in a way that regulates signal peptide insertase activity of TatC. **(A)** The TatB-TM prevents access of the signal peptide to its transmembrane-oriented binding site at TatC. Color code: light pink, TM1-4 of one TatC; salmon pink, transmembrane signal peptide binding site; red, bound signal peptide; green, TatB that blocks access of the signal peptide c-region to TatC; blue, APH of neighboring TatB that covers the bound signal peptide. **(B)** The TatB-TM is tightly bound by TM5 of the TatC TM5/TM6 domain in the complex. Color code like in **(A)** but with a second TatC included (light beige: TM1-4 and TM5/6-domain in orange). **(C)** The TatC TM5/TM6 domain with its associated TatB is positioned deep in a molecular cage generated by the TatC TM1-4 of the three subunits. **(D)** View from the cytoplasmic side into the cup-shaped cavity formed by the TatBC complex, highlighting the three TatC TM5/TM6 domains that are tilted into the cavity. Images of the molecular structures in Figures 2–4 were generated by use of ChimeraX 1.10.1 16. Color code for **(C)** and **(D)** like in **(B)**, but with the third TatC and TatB included in gray.

The twin-arginine motif of Tat signal peptides is bound by a cavity formed by one TatC and two TatB 9 (Figure 1**B**). The site is covered by a TatB amphipathic helix that must be lifted if Tat substrates enter the binding sites laterally from the membrane, as shown for selected *E. coli* and *Streptomyces* proteins 21, 37. In case of the Rieske protein of *Streptomyces*, this is a polytopic membrane protein with an internal signal peptide that mediates Tat transport of a C-terminal domain, which proves that there exists a lateral entry for membrane-associated Tat substrates to the twin-arginine binding site in addition to a possible direct binding to TatBC 21. TatC alone has a signal peptide insertase activity and consequently possesses a large signal peptide binding site that is crossing the membrane 35. Such a signal peptide binding site has been postulated also by the research group of Bil Clemons, based on the crystal structure of a TatC monomer 38. As the twin-arginine motif remains bound at its recognition site throughout transport 36, the hydrophobic (h)-region of the signal peptide must have access to a binding site on the TatC surface that reaches from the twin-arginine binding site at the cytoplasmic side of TatC TM1/TM2 to the extracytoplasmic side of these helices. There is ample *in vitro* and *in vivo* cross-linking data showing that TatC interacts with the entire signal peptide, including the h-region 10, 39. However, as seen in the substrate-bound structures 8, 9, the highly tilted N-terminal helix of TatB blocks the access of the signal peptide h-region to its binding site at TatC in the substrate-bound structures (Figure 2**A**). This explains the observation of Fröbel et al. that TatB blocks the TatC insertase activity 35. A twin-arginine-dependent catalytic signal peptide insertion activity of TatC makes only sense if it is related to the transport of Tat substrates. To function in membrane insertion of the signal peptide in the TatBC complex, the N-terminal TatB helix therefore needs to be moved away from this buried position in a step following signal peptide binding. The N-terminal helix of TatB interacts tightly with TM5 of the TM5/TM6 helix bundle of a neighboring TatC (Figure 2**B**). This whole helix bundle is highly tilted and connected to TM1-4 via a flexible linker sequence. The extremely tilted TatC TM5/TM6 protrude, together with the associated TatB N-terminal helix, into a cavity of the TatBC complex (Figure 2**C,D**). This cavity is formed by TM1-4 of the three TatC protomers, which stably connect at the top of the complex and build a firm cage structure (Figure 2**C**). In summary, the TatBC complex forms a cage by helices 1–4 of the three TatC subunits. These helices are connected by a flexible linker to strongly tilted helices of TatC TM5/6 and the TatB N-terminus, which together protrude into the central cavity of the cage.

### Biochemical and structural evidence for a signal peptide-induced domain movement in TatBC

**Figure 3:**
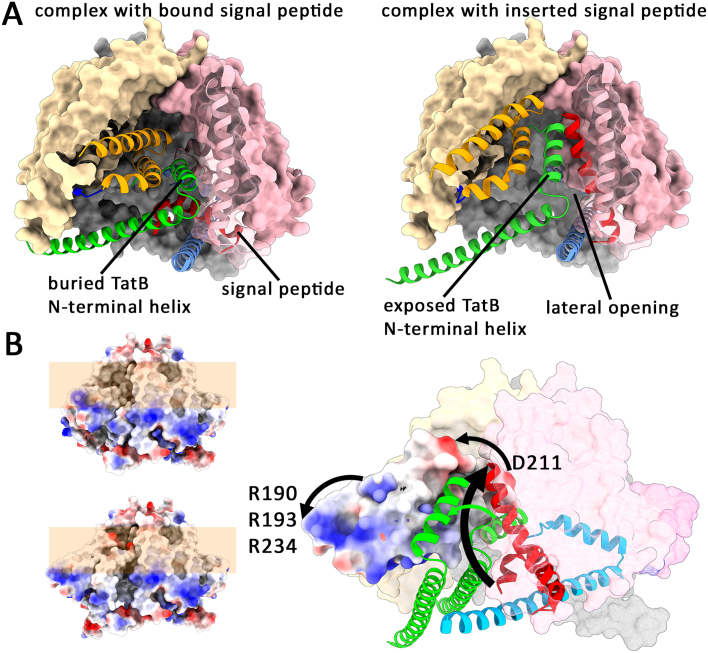
Signal peptide insertion and its energetization. **(A)** A likely movement of the TatC TM5/TM6 domain can position TatB on the surface and permit access of the signal peptide h-region to TatC, resulting in signal peptide insertion (see supplemental Video S1 for clarity). Color code as in Figure 2. **(B)** The movement can be energized by the electric field at the energized membrane. Especially a conserved aspartate of TatC (in *E. coli* D211) and a patch of arginines trigger the movement. The reason for its essential role was unclear so far. The surface is colored by electrostatic potential (red: negative; blue: positive), either for the complete surface (small structures on the left) or specifically for one moving TatC TM5/6 domain (large structure on the right).

As the N-terminal helix of TatB has to move away in order to permit access of the signal peptide h-region to its TatC binding site, we postulate that the TatC TM5/6 helix bundle must move together with the bound TatB. The necessary movement at the hinge between TM4 and TM5 of TatC exposes the TatB N-terminal helix at the surface and forms a lateral opening and exit site for the inserted signal peptide (Figure 3A and Suppl. Video S1). When the TatBC complex laterally opens by the domain movement, vacant hydrophobic surfaces inside need to be occupied by membrane lipids. Such lipids have been identified in the structure 9, and are likely needed to prevent a destabilization of the translocon. Accordingly, phosphatidylethanolamine has been identified as being essential for Tat transport 40. The movement-induced exposure of the transmembrane-helix of TatB to the surface would generate a potential docking site for TatA in close proximity to the TatC TM5/TM6 domain. This is supported by the observation that TM5 contacts TatA under conditions of translocon saturation 31. Other evidence comes from *in vitro* studies by Fröbel et al., who observed TatBC, twin arginine- and pmf-dependent cross-links of Tat signal peptides to a single TatA 41. These crosslinks can be explained by a placement of the inserted signal peptide in the lateral gate at the moved TatC TM5/TM6 domain in close proximity to TatA. The absence of additional TatB or TatA crosslinks supports the view that the *E. coli* TatB exchanges with TatA during transport, and thus such a transient exchange can be required in three-components Tat systems for efficient initiation of translocation. A permanent exchange is unlikely though: Roland Freudl and coworkers found that single mutations in the N-terminal 6 residues could transform an *E. coli* TatA into a TatB 42. The corresponding region in TatB is exactly the region that contacts the “periplasmic” TatC loop between TM5 and TM6 that is positioned deep in the complex in the resting state. This makes sense, as the N-terminal 6 residues of TatB are designed to stabilize the interaction that is required for the movement of TatB with the TM5/TM6 domain – and thus TatB must be bound to the TatC TM5/TM6 domain in the resting state, not TatA.

The conformational switch of the tilted helices would thus explain (i) the signal peptide insertion, (ii) the TatA mutations that render TatA bifunctional, (iii) the formation of a TatA recruitment-site at the complex surface, (iv) the transport-enhanced cross-links of TatC TM5 with TatA, and (v) a lateral opening at the translocation site that would be required for the exit of the inserted signal peptide. But there is also experimental evidence for this key conformational switch coming from the structural analyses: The N-terminal helices of the TatB subunits become more flexibly positioned inside the complex in response to signal peptide-binding (Figure S8g in 9). This indicates a higher flexibility of the TatB and the tightly interacting TatC TM5/TM6 domain, which are hence able to move in response to signal peptide-binding. The movement explains that signal peptide-binding triggers the recruitment of TatA oligomers already without globular mature domains of folded substrates, answering the key question of how TatA is recruited to the translocation site 17.

### The domain movement requires membrane energetization

But what would energize the domain movement and why is there no CryoEM structure of a signal peptide-bound TatBC complex with a switched conformation? The answer to these questions can be found in site-specific cross-linking studies: The complete signal peptide interacts with TatC under translocation conditions 39. However, while only the twin-arginine motif interacts with TatC already in the absence of the proton motive force (pmf) 10, 36, the interaction to the h-region requires a pmf 43. In agreement with the model and the structure is also the fact that TatB interactions of the h-region do not depend on a pmf: As TatB hinders access of the h-region to TatC in the resting state prior to the domain movement, the h-region contacts TatB in the presence of CCCP that blocks the domain movement. The membrane energetization has long been shown to be essential for Tat-dependent protein transport 44–47. The research group of Siegfried Musser found that there is a ΔΨ -dependent step during Tat transport, and he suggested that “… instead of being consumed to energetically drive transport, it is possible that the ΔΨ is instead coupled to protein conformational changes (“gating reactions”) that are required for transport”. and “However, a reasonable scenario is that movement of certain charged regions within Tat proteins could be induced by a ΔΨ ”. 48. Consistent with this idea, the highly tilted TatC TM5/TM6 domain has a conserved negatively charged spot on the “periplasmic” end 38, which is located deep in the membrane, thus enabling the use of the electric field for the movement of the domain into a conformation that places the negative charge closer to the periplasmic surface of the membrane (Figure 3A,B). In *E. coli*, this residue (D211) has been demonstrated to be absolutely essential for Tat transport 49. The movement could in principle be enhanced by TatB E8, which is even deeper positioned in the membrane and could similarly move toward the positively charged side of the energized membrane. This may be a reason for the conserved occurrence of glutamic acid in TatB and bifunctional TatAs of some phyla at this position. In addition, often a positively charged spot of exposed arginines on the cytoplasmic face of the TM5/TM6 domain is embedded into the membrane and likely supports the domain movement (Figure 3B). As this energetization is abolished by any membrane protein solubilization, sample preparation for CryoEM automatically locks the complex in the conformation prior to the switch, irrespective of substrate-binding, which is why CryoEM could not show the outlined domain movement.

### Upon signal peptide insertion, the globular domain relocates to the translocation site

To pass through the membrane, the globular domain of the Tat substrate needs to be positioned to the translocation site close to the lateral opening generated by the movement of the TM5/6 helix bundle. In the CryoEM structures that show the position prior to the movement, the globular domain is located at a more central position of the *cis*-side (in prokaryotes the cytoplasmic, in plant plastids the stroma, and in mitochondria the matrix side), making contacts to more than one TatB amphipathic helix 8, 9. This agrees with the crosslinking data of the research group of Matthias Müller 50. At this time point, the signal peptide reaches the twin-arginine motif binding site via a cavity underneath a TatB amphipathic helix. As three amphipathic helices form a closed triangle, this triangle requires opening to permit the passage of the mature domain to a lateral translocation site close to the signal peptide insertase site at TatC TM1/TM2. As already mentioned in the context of substrate-binding above, the amphipathic helix cannot be firmly bound, and thus the TatC TM5/TM6 domain movement with the bound TatB adjacent to the signal peptide insertion site will open the triangle formed by the TatB amphipathic helices. In other words, the TatC TM5/6 domain movement not only activates signal peptide insertase and generates a lateral opening that permits later TatA assembly and signal peptide release, it also enables the relocation of the globular domain of the substrate to the lateral opening of the TatBC complex. At the end of this phase I, the signal peptide is inserted, the N-terminal region of the mature protein is unfolded and pulled through the membrane by this insertase activity, and the globular domain is positioned on the cis site of the membrane at the lateral opening of the TatBC complex. This intermediate was first identified by the research group of Ralf Bernd Klösgen, who termed it “translocation intermediate 1” or “Ti-1” 51, and it is the pmf-dependent state in which the substrate is deeply bound in the TatBC complex, as identified by Gerard and Cline 52.

## PHASE II OF TAT TRANSPORT: DOCKING OF TATA, MEMBRANE DISRUPTION AND DIRECTIONAL TRANSLOCATION

### Previously unanswered key questions that helped to understand TatA-dependent translocation:

- How is it possible that unfolded linkers between the signal peptide and the globular domain are tolerated by the Tat system, as observed by Cline and McCaffery as well as by us 53, 54
- What are the structural characteristics of the ordered cluster of at least 16 TatA protomers that is generated at TatBC upon RR-dependent binding of the signal peptide to TatBC, as observed by Carole Dabney Smith et al. 17?
- Why does substrate-interaction with TatA shift the hinge region of TatA into a fully solvent-exposed position, as shown by the research groups of Ken Cline and by us, and why is this transition not TatBC-dependent 23, 55?
- Why does substrate-interaction with *E. coli* TatA quantitatively protect a specific region of the TatA amphipathic helix in a TatBC-dependent way, as shown by us 23?
- Why do large TatA assemblies diffuse either slowly, as expected for membrane-integral proteins, or rapidly, as expected for membrane-associated proteins without transmembrane helix, as shown by Yves Bollen et al. 56?
- What is the energy-demanding step in phase II of Tat transport 48?

### A simple pulling of the folded protein through a disordered TatA assembly cannot explain Tat transport

When the TatC TM5/6 domain movement exposes the bound TatB at the surface, a docking of TatA to the translocation site starts, likely by an interaction with TatB and/or with its binding site at TM5. At least 16 interacting TatA protomers are recruited, and their interaction changes in a way that their C-terminal regions can be readily cross-linked 17. It has been reported that TatA assemblies can weaken the membrane by local thinning and lipid disorder, which is currently believed to permit the passage of folded proteins next to TatBC complexes 25, 27, 57, proposed to be energized by a TatC-dependent pulling 22.

However, although the above described signal peptide insertion by TatBC exerts a force that pulls the N-terminal region of the folded domain into the membrane, this one pulling step cannot be responsible for the translocation of the folded protein: The plant Tat system has been shown to translocate small folded proteins that are connected to the signal peptide via an unfolded linker of at least 75 residues (more precisely, 15 × GGGGS repeats) 54. Similarly, we discovered that the *E. coli* Tat system is able to translocate unfolded linkers of 110 residues together with a C-terminally fused tightly folded ca. 10 kDa globular domain 53. In addition, it has been shown that two globular proteins fused by a linker can be translocated by the Tat system 58. These studies indicate that Tat substrates do not pass through the membrane via a single pulling stroke, and as the twin-arginine motif can remain bound to the same binding site until translocation is completed 36, there must be a second phase in Tat transport that completes the process after signal peptide insertion. This is further supported by bioenergetic analyses that point to a second, longer period of membrane energetization that is required to complete transport *after* phase I, which we know now is the above described ΔΨ -dependent signal insertase activity 48. But what is the nature of this “phase II” of transport, in which TatA completes the translocation process?

### TatA likely forms a more defined structure to permit Tat transport

The association of TatA can either be unordered or ordered. We originally proposed unordered TatA arrangements when we invented the idea of membrane-weakening in 2003 22. Interacting flexible TatA transmembrane-helices and amphipathic helices could weaken the membrane, as experimentally shown 23, 57, and TatA transmembrane-helices and amphipathic helices would need to be somehow pushed away by the membrane-crossing globular Tat substrate. An imposed directionality could be achieved by the fact that the signal peptide C-region is already translocated across the bilayer at this time point, and there is some energy provided by the refolding of the N-terminus of the globular Tat substrate that had been unfolded in the course of signal peptide insertion. However, this folding energy cannot be strictly required and it also cannot be a simple pulling mechanism, since fully folded proteins can be translocated that are bound to signal peptides via long unfolded linkers 53, 54. Without more energy, it is difficult to explain how interacting TatA helices and lipids can be reliably pushed away during transport. TatA obviously requires TatBC for translocation, and therefore at this phase II of transport there needs to be some contribution of TatBC other than pulling through a TatA assembly. In addition, if unordered TatA assemblies would catalyze a membrane passage, topology changes would not be controlled and TatA/substrate interactions during transport could generate irreversible holes that would harm the cells just like some antimicrobial peptides. Furthermore, if any kind of pulling or pressing would be responsible for the directionality of transport through TatA assemblies, the TatA assembly would need to be somehow in a fixed position relative to TatBC during translocation, as a highly flexible TatA assembly could move with the membrane without permitting a passage through the membrane. Moreover, it is hard to imagine that anything specific could be triggered by a pmf in an unordered TatA assembly, but phase II of Tat transport requires at least a low pmf 48. Based on this line of argument, and given a much better alternative (see below), we exclude that an unordered assembly of TatA generates the pathway through the membrane.

### TatA forms rings that could allow a controlled TatBC-dependent membrane perforation

An alternative to an unordered TatA cluster is an ordered, likely ring-shaped TatA complex that encloses a defined weakened membrane area. The membrane-weakening would require that the short transmembrane-helices are flexibly connected to the rigid structure, pointing to the center of the enclosed membrane area. It has been shown by crosslinking analyses that substrate-binding to TatBC induces an ordered interaction of multiple TatA protomers, which without substrate-binding interact differently 17, 59. A well-defined ring with identical TatA protomer interactions would best explain such a regular crosslinking and combines this with advantageous properties. In the already thinned membrane environment of the TatBC complex 9, docking of such a ring to TatBC could extend the thinned membrane area that surrounds TatBC into the inner space of the ring. Beyond thinning, the membrane would be further destabilized by the hydrophilic N-terminus of the substrate’s mature domain that crosses the membrane as a result form signal peptide insertion.

TatA can in principle form rings: When TatA was solubilized, purified and analyzed by electron microscopy, it was found to form rings of variable diameter 60. Interestingly, the central pore of these rings was covered only on one side by electron density. The authors believed at that time in a gated pore mechanism and interpreted this electron density as a lid on the cytoplasmic side. The lid-idea was later discarded, and the rings were never understood. Note that smaller rings may have been generated during solubilization due to detergent sensitivity of larger rings, which can also explain the observation of on average smaller rings of highly similar structure for TatA ${}_{d}$ from *B. subtilis* 61. In another study, we found that with mild overproduction of TatABC, TatA formed hollow tubes with an inner diameter of ca. 6.7 nm and an outer diameter of about 11.5 nm, which were visible by electron microscopy in thin sections of cells after ultrastructure-preserving cryofixation and cryosubstitution 62. These tubes had the dimensions of the rings reported before, and they appeared like stacks of multiple rings. Importantly, their formation depended on TatBC and therefore they originated at membranes. In the absence of TatB, the tubes were not hollow and contained a central, electron-dense region, indicating that TatB affected the TatA rings in a way that resulted in a hollow central space 62. This hollow space was large enough to accommodate the largest known Tat substrates, such as the heterodimeric FdnGH of formate dehydrogenase (142 kDa, ca. 6.4 nm, PDB1KQF, 63). In summary, TatA has the ability to assemble to ring structures of sufficient size to serve for translocation of folded proteins, even with more than one subunit.

### AlphaFold 3 predicts TatA rings that explain so far not understood findings

**Figure 4:**
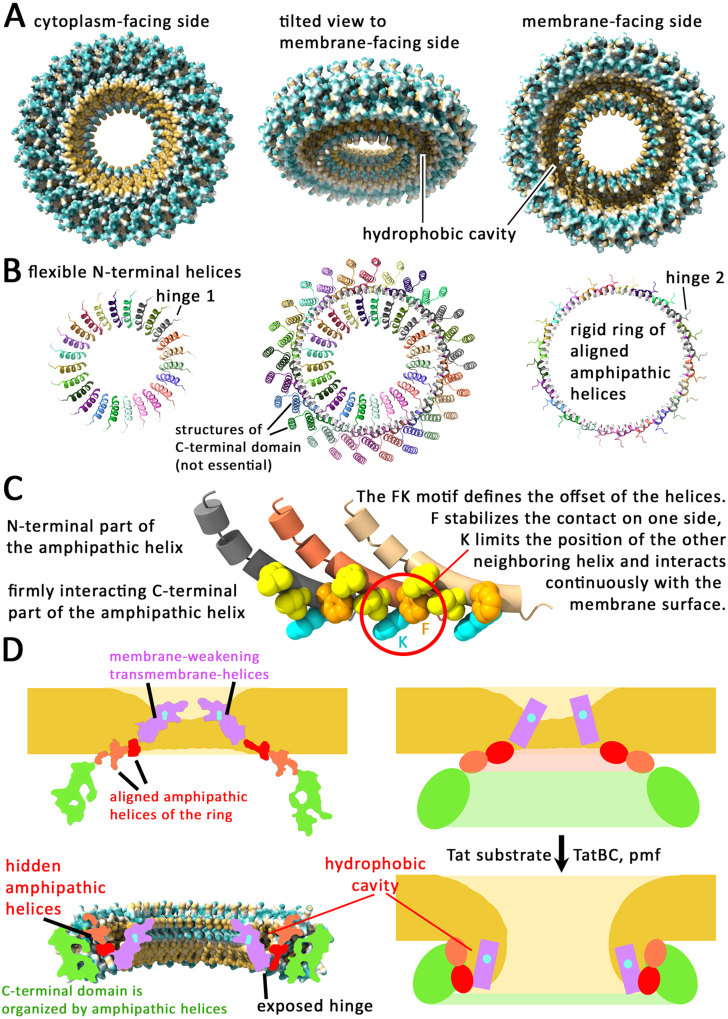
AlphaFold 3 (AF3) predicts a ring structure for TatA that makes physiological sense and that explains the importance of conserved TatA residues. **(A)** AF3 predicts a ring structure for TatA in which the short transmembrane domains are positioned in the inner side of the ring. Shown is a 24mer of full-length *E. coli* TatA with its surface colored by hydrophobicity (cyan, hydrophilic; beige, hydrophobic). Note the hydrophobic cavity that is formed by the amphipathic helices and the transmembrane helices of the ring. **(B)** Ribbon structure of the ring, individual chains colored differently, highlighting the essential regions for TatA functionality, which are the transmembrane helix and the amphipathic helix. Note that the amphipathic helices are tightly interacting and form a stable ring, whereas the transmembrane helices are bound to the ring via the flexible hinge1 region. Note also the presence of a second hinge at the other end of the amphipathic helix. The two hinges can allow orientational changes to place the transmembrane helices in the membrane. **(C)** The amphipathic helices interact in an ordered way that explains the important roles of the essential FK motif. Shown are the amphipathic helices of three interacting TatAs in individual colors (gray, ochre and light beige), the conserved F (orange) and K (cyan) near the end of the amphipathic helix and the hydrophobic residues (yellow) that interact with the conserved F to form the regularly staggered helix interaction. **(D)** Scheme of the two states of TatA rings that likely play a role for Tat transport, based on the AF3 structure, which explains TatA functionality. A cross section of an *E. coli* TatA ring is shown at the bottom left and the upper left shows cross section areas of a hypothetical membrane-inserted state of this ring. Green, cross section area of the C-terminal domain (which is likely largely unstructured); orange/red, cross sections of interacting TatA amphipathic helices; purple, transmembrane helix cross section. On the right side, the structures are more schematically shown, for visualization of the membrane weakening (upper right scheme) and the membrane disruption (lower right scheme). The color code is the same as on the left side. The weaker colored thick green, reddish and beige lines provide a sense of the circularly arranged TatA protomers and the generated membrane hole.

To function, the signal peptide release site of the TatBC complex requires to open towards the TatA ring, which is why TatBC needs to be integral part of the rings that are present at active translocons. According to cross-linking data, at least 16 subunits reorganize at the substrate-bound translocon 17. A closed ring of 22–26 TatA subunits would be in the range of the dimensions of tube cross-sections or solubilized *E. coli* TatA rings 60, 62. AlphaFold 3 (AF3, 15) models such TatA rings with amphipathic helices that interact in a stable closed circle that is the basis for the ring (Figure 4A). The short transmembrane helices are seen in the center of the rings, which could easily insert into the membrane, as the amphipathic helices are preceded and followed by hinge regions and are thus flexible in their orientation (Figure 4**B**). After membrane insertion, the complex would have exactly the shape of the previously reported TatA rings, but what has been originally interpreted as a lid on the cytoplasmic side was likely the bundle of transmembrane-helices, and what has been assigned as transmembrane region were the polar and amphipathic TatA domains on the membrane surface 60.

The key features of the AF3-predicted rings, which are the staggered interacting amphipathic helices and the inner circle of transmembrane helices, apparently are conserved across phyla from archaea and bacteria (Figure S1). The pTM and ipTM scores for the optimum ring sizes range from ca. 0.2 to 0.6, depending on the specific sequence, with a tendency for higher scores in case of extreme thermophilic TatAs (Figure S1). In case of “normal” membrane protein complexes, scores in this range would be evidence against the validity of such models, but in case of TatA, scores are expected to be lower than found for stable protein complexes, because (1) TatA rings have a very unusual membrane-association and likely require a membrane environment to be formed, which is not integrated into AF3 calculations, (2) they need to assemble in the presence of membrane-inserted substrate (which cannot be included in AF3 calculations), (3) they need to disassemble after transport and thus cannot be per se stable, and (4) they often have large highly charged C-terminal regions of score-lowering uncertain and variable predicted structures that may be unstructured in most cases, such as found for TatA ${}_{d}$ from *B. subtilis* and TatA from *E. coli* 64, 65. APH rings can have predicted Local Distance Difference Test (pLDDT) scores of 70–90 and can therefore be considered to be of high confidence and generally reliable (Figure S1). As the architecture of the rings is conserved and found even in the models of lower-scoring TatAs, we postulate that the basic ring architecture is likely correct and a common characteristic for all TatAs. As outlined below, the ring structures can explain the conservation of important residues and yet unexplained results of molecular biological and biochemical studies, which is – beside the conservation from archaea to bacteria – the main reason why we believe that the AF3-predicted rings must strongly resemble the translocon-associated structure in the substrate-associated conformation: (i) The AF3-predicted ring attributes an essential role for ring formation to the amphipathic helix, and the amphipathic helix is known to be essential for Tat transport 3. (ii) The AF3-predicted ring explains the high conservation of the FK-motif (in plants and cyanobacteria FQ motif) in the amphipathic helix (in *E. coli* F39/K40, 66), as this motif determines the staggered arrangement of the interacting helices that results in the defined ring: K interacts with the phospholipid membrane surface and positions the end of the interacting amphipathic helix at its side (Q in plants and cyanobacteria that have glycolipids), and the preceding F has the tightest interaction with the neighboring helix (**Figure 4C**). This is experimentally confirmed, as TatA-F39A mutation results in irregular and inactive TatA assemblies, indicating that firmly ordered amphipathic helices are essential for functionality 33. (iii) The AF3-predicted ring also explains the dominant negative phenotype of the F39A mutation, as all subunits of the ring require F39 to correctly interact with neighboring subunits 67. The alignment and orientation of the amphipathic helices in the ring would also fully agree with biophysical analyses that indicated such an ordered and tilt orientation 68. As seen below, the amphipathic helix interactions are of key importance for our proposed mechanism.

### The predicted TatA rings suggest a membrane disruption mechanism that is experimentally supported

Although details of the regular interaction of the amphipathic helices in the predicted TatA ring are in astonishing agreement with available experimental data, which allows the assignment of functions of important conserved residues, the orientation of the amphipathic helices and the position of the transmembrane helices is surprising. In the predicted structure, only the C-terminal end of the amphipathic helices could make contacts to the membrane surface, with the conserved lysine of the FK motif being the key residue, while the hydrophobic side of the amphipathic helices hardly contact the membrane surface and instead mediate helix-helix interactions and form a hydrophobic cavity together with the N-terminal transmembrane-helices (Figure 4A-C). This is possible, because the tilt orientation of the helices positions the N-terminal transmembrane helices inside the ring. Surely, this positioning of the transmembrane helices is influenced by the absence of membrane constraints in AF3, but the structure indicates that a ring of amphipathic helices can be formed that places the transmembrane helices in its inner surface. To comfortably insert the transmembrane-helices into the membrane, the amphipathic helix would need a reorientation and/or a kink. Such a kink could be formed at the conserved internal flexible positions of the amphipathic helix (in *E. coli* G34), and also a reorientation could be easily achieved, as the amphipathic helices have flexible linkers at both ends (Figure 4D). Therefore, the AF3-predicted ring could in principle insert into membranes. In the membrane-inserted state, the presence of multiple aligned amphipathic helices and likely disordered transmembrane helices would weaken the drag of the membrane, like proposed earlier 22.

As reorientation of the amphipathic helices thus has to be postulated to explain the membrane integration, we considered that this conformational transition might also function in the opposite direction: The short TatA transmembrane helices could be in principle pulled out of the destabilized membrane *into* the ring where they align at its inner border as seen in the AF3 structure. As the tilted amphipathic helices generate a large circular hydrophobic cavity with the pulled-out transmembrane helices, the interacting lipids of the destabilized bilayer in the center of the ring would be pulled into this cavity, resulting in a transient membrane disruption and formation of an aqueous channel (Figure 4D). The cavity – as modeled by AF3 – certainly reflects an energetic optimization and shielding of hydrophobic surfaces, but it is important that the structure can in principle form a space for lipids during membrane disruption, and the formation of this structure will likely be facilitated by the lipids. After this switch, the TatA ring would be transformed from an integral membrane protein to a membrane surface-attached ring. Note that the transmembrane helices have been found to have an intrinsic ability to flip out of the membrane core, which has been suggested by the research group of Anne Ulrich to play a key role in TatA functionality 69. This finding is in full agreement with our proposed mechanism of a TatA-mediated membrane disruption.

As at this time point the Tat substrate is already anchored to the trans-side of the membrane by its inserted TatBC-bound signal peptide, the folded protein can rapidly diffuse through this open pore. Only this open pore explains how long linkers between signal peptides and globular domains can be tolerated 53, 54. The efficiency of directional transport will gradually decrease with linker-length: As the signal peptide insertase activity of TatBC unfolded the N-terminal region of the mature domain, this region will fold again at the moment when the lipids are removed, thereby pulling the mature domain to the trans-side. The contribution of this pulling to directional transport will be abolished by linkers between the signal peptide and the globular domain. Importantly, this pulling is not essential for transport by our proposed mechanism, but only globular proteins will be transported efficiently. In agreement with this finding, it has been clearly demonstrated that unfolded artificial Tat substrates translocate progressively less efficiently as the length of the unfolded region increases 70. The cytoplasmic turgor in bacteria and archaea will result in a solvent flow through the pore and thus may accelerate the process of translocation of folded proteins, but this solvent flow cannot be essential because thylakoids or mitochondria have no cell wall and thus no significant turgor.

The membrane is locally depolarized when the pore is formed, and therefore even Tat substrates with high isoelectric points are accepted. A transient local depolarization definitively takes place, as transport of each Tat substrate has been shown in the plant Tat system by the group of Steve Theg to be accompanied by the leakage of an estimated ca. 79,000 protons, which supports the membrane-disruption mechanism 71.

In summary, the details of the AF3 structure gave us the idea of a mechanism that involves a controlled transient membrane disruption, which can explain the so far unexplained translocation of linker-containing Tat substrates. We believe that the diverse structural and functional details that perfectly fit to this idea cannot be coincidental, and the ring also explains many other open questions, as outlined below.

### The membrane disruption mechanism explains the TatBC dependence and the 2nd energy requirement

To disrupt the weakened membrane bilayer in the TatA ring, the amphipathic helices need to change their conformation in a coordinated and TatBC-dependent way. The main reasons for the involvement of TatBC are likely (i) a required specific signal peptide insertion for the proteins that need to be translocated, and (ii) a required energetization and organization for the concerted conformational change of all TatA amphipathic helices in the ring. The signal peptide insertion is needed to anchor the transported protein to the trans-side, and the twin-arginine motif therein serves as binding site for the signal peptide insertase and as determinant for the selection of Tat substrates. The energetization is needed because TatA rings exist in two states – membrane-anchored and membrane-extracted – and there is an energy barrier to overcome the transition between these two states. TatA is a protein at the membrane surface that is only membrane-anchored with one short transmembrane helix that evolved to be extractable, and thus TatA alone has no way to couple membrane energetization to a conformational change, which is why we think that TatBC needs to energize this step.

Two of our studies showed an influence of TatBC on TatA topology that we could not explain so far: From a study that compares the substrate-induced effect on the TatA amphipathic helix orientation in the absence and presence of TatBC, we learned that substrate binds to TatA already in the absence of TatBC, causing TatA conformational changes, but only in the presence of TatBC the region G33-S35 becomes inaccessible by substrate-binding, showing that TatBC specifically influences the TatA transition upon substrate-binding 23. This could be explained by the AF3-structure, as access to this TatA region is hidden by helices that can be formed by the C-terminus of TatA. This is another evidence that the AF3 model resembles the transport-induced structure. In the absence of TatBC, the G33-S35 region is always exposed – irrespective of substrate-binding. This is also supported by a study that showed a transport-dependence of TatA-TatA interactions in the C-terminal domain of TatA 59, and by another study that demonstrated a reduced accessibility of central and C-terminal parts of the TatA amphipathic helix during active transport 55. In conclusion, without active transport, the C-terminal domain does not fold like seen in the AF3 structure and the amphipathic helix changes its environment, which indicates that transport is achieved by a structural change of TatA rings at substrate-bound TatBC complexes.

If a binding site is transiently generated for the TatA conformational transition that results in membrane disruption, then this should be the exposed TatB binding site or TatB itself that in phase I has moved to the lateral opening, which depended on the electric potential. In our model, the domain movement placed the binding site with the bound TatB half-way out of the inner surface of the membrane (Figure 3**B**). It is possible that TatB has to be transiently released from TatC TM5 to permit an optimum TatA interaction. In this case, TatB might remain in the near, possibly anchored by interactions of the amphipathic helices. Alternatively, TatB can mediate the TatA interaction without being released from TM5. The interaction of the TatA ring with the TatBC complex can fulfil two important functions for Tat transport: (i) it can open the TatA ring at the lateral gate, permitting the passage of the mature domain N-terminus that was membrane inserted during signal peptide insertion, and (ii) it can trigger the concerted extraction of the ring, which would disrupt the membrane and generate an aqueous hole.

At the TatBC complex, there is no evidence for energetization-dependent movements other than the described movement of the TatC TM5/TM6 domain and the reorientation of the internal TatB to the surface. The reason for the pmf-requirement of phase II may thus be an effect of the pmf on the TatA ring structure per se (*i.e.* the positioning and orientation of the TatA domains) and/or the need to maintain the moved TatBC complex domain stably exposed at the surface until the correct docking to the TatA ring is accomplished. Minor movements back into the interior of the TatBC complex must be prevented, which would make the crucial TatA interaction impossible, and the formation or opening and lifting of the ring onto the membrane surface could not take place. As an alternative to a more active lifting of the TatA ring by TatBC, it might be that simply the docking-caused ring-opening and local membrane defects at the docking site, possibly related to the unfolded N-terminal end of the globular domain, trigger the concerted extraction of the TatA transmembrane helices into the inner surface of the ring. The result would be in both cases a lifted (not membrane-integral) ring on the membrane surface that surrounds a locally disrupted membrane (Figure 4**D**).

In this context it is interesting to note that the membrane-stress response protein PspA of *E. coli* and its homolog LiaH in *B. subtilis* have been found to interact with Tat systems, which can form scaffold-like structures in conjunction with TatA that might help to suppress any potential extensions of membrane disruptions or membrane perturbations, or prolonged unspecific molecular leakage across the membrane 72, 73.

### Experimental evidence for a transient lifting of TatA rings onto the membrane surface

The two above-described states of the TatA ring – membrane-inserted versus membrane-attached – explain the finding of the research group of Yves Bollen and Holger Lill that large TatA complexes switch between fast and slow diffusion 56. In that study, it has even been stated that the fast diffusion is too fast for a trans-membrane protein complex consisting of multiple TatA monomers. Based on these findings, they proposed “…that TatA complexes switch between a slowly diffusing transmembrane conformation and a rapidly diffusing membrane-disrupting state that enables folded proteins to cross the membrane…” 56. The Tat mechanism that we propose fully supports this finding. The two states of TatA complexes are also consistent with the fact that about half of the TatA protomers can be stripped off from membranes by carbonate washes, whereas the other half cannot 23.

### Priming of TatA rings by bound substrate, and limited time window for membrane disruption by TatA

The effect of membrane-weakening is enhanced by substrate-association with TatA, indicating that the globular domain of the protein substrate itself influences the TatA-mediated destabilization 23. The enhancement of membrane weakening likely involves above-mentioned orientational changes of the substrate-associated TatA amphipathic helices 23, 55. The substrate interactions with TatA are important, as they increase translocation efficiency 39. For transport, it is crucial that the associated TatA ring is attached to TatBC, as the substrate must be translocated while the signal peptide is still bound to TatC. Otherwise, the diffusion through the pore would be undirected. Interestingly, we have observed that Tat substrate interactions with TatA result in much of the conformational changes already in the absence of TatBC, suggesting that substrate-interaction prime the TatBC interaction 23. Especially the important movement of the hinge region to the surface of the TatA complex is already accomplished without TatBC, which reflects the transition of the amphipathic helix to a straight conformation that is seen in the AF3 structure. This suggests that TatA forms rings already in the absence of TatBC, and that substrate-binding thus already puts the transmembrane helices under tension, with the amphipathic helices on the membrane surface being reoriented and ready to interact with TatBC, and only a minor TatBC-dependent event is required to initiate the extraction of the transmembrane helices out of the membrane. This is further supported by the fact that substrate-binding already results in increased TatA-dependent proton leakage in the absence of TatBC 23.

It is likely that substrate-binding induces the TatA ring formation, as the *in vitro* translated and membrane-inserted TatA was quantitatively transformed into the hinge-exposing TatA upon saturating the system with substrates, and the surface-exposed hinge is a characteristic of the ring structure 23. Substrate- and transport-dependent crosslinking of at least 16 TatA subunits with double cysteine exchanges was achieved with positions that interact in the C-terminal TatA region 17. If the C-termini of TatA protomers have predominantly unstructured regions, as supported by structural analyses 64, 65, the likelihood for catalyzed disulfide formation between neighboring unstructured domains is high, which explains the efficiency of disulfide formation in that region after ring formation. Disulfide-crosslinking with double cysteine exchanges in the flexible transmembrane helix was also enhanced under transport conditions, with cross-linking efficiencies at varied cysteine positions indicating more torsional flexibility than found in other membrane proteins 17. Disulfide cross-links with single cysteines were less efficient but similarly could be enhanced by transport conditions, especially in the amphipathic and C-terminal regions of TatA, pointing to an increased proximity of these domains during transport 59.

After transport, the signal peptide is in a membrane-spanning N-inside/C-outside orientation, as known for Tat transport 74, and thus anchors the folded Tat substrate to the trans-side of the membrane, an intermediate state that has been identified as Ti-2 in *in vitro* studies of the plant Tat system 75. The membrane pore needs to close by influx of lipids from the surrounding. The lipid-contacts of the amphipathic and transmembrane helices likely prevent that membrane disruption can continue beyond the inner area of the ring. Tat transport is thus a spatially controlled perforation of the membrane.

As the membrane disruption is transient, there will be only a short time window for the passage of the folded substrate through the transient pore that is formed by a TatA-dependent membrane-weakening and -disruption. In case of a delay of transport due to larger unfolded regions and/or exposed lipid-interacting hydrophobic regions of the substrate, membrane sealing will prevent translocation in phase II without blocking the Tat system, as phase I is completed and the signal peptide released from TatBC into the membrane. In agreement with this prediction, we showed already that targeting of unfolded, not translocated Tat substrates to the translocon does not block translocation 76. The completion of phase I and a failure of phase II explains also that what has been termed “large scale translocation reversal” in a study of the plant Tat system in which signal peptides were Tat-dependently cleaved without successful translocation of an artificial cargo protein 77.

## PHASE III: CLOSING OF THE CATALYTIC CYCLE AND RESETTING OF THE SYSTEM

### Release of the signal peptide into the membrane, and reset of the TatBC complex

Any transported Tat substrate is initially captured in the TatBC complex. According to studies of the Musser-group, the release of the Tat substrate from the Tat system “requires a significant fraction of the total transport time” 78. The movement of the TatC TM5/TM6 domain creates a lateral opening for signal peptide release, and it is likely that an additional movement of TM1 next to the twin-arginine motif binding site facilitates signal peptide release, just as this flexibility can also contribute to the accommodation of the signal peptide and the unfolded N-terminal region of the substrate. Note that beside TM5/TM6, also TM1 has been predicted by the group of Bil Clemons to be flexible 38. As long as the signal peptide is not released from the TatBC complex, the TatC TM5/TM6 domain with its associated TatB cannot switch back into the resting position. As the initial movement of the domain depended on the membrane energetization, the movement back to the resting position is likely facilitated by the local depolarization of the membrane. Once the complex is in the resting state, membrane energetization can build up again to energize the next translocation.

### Reset of TatA rings to membrane-inserted clusters that can interact with Tat substrates

After transport, the TatA ring is released from the translocation site, because the TatC TM5/TM6 domain with its bound TatB re-inserts into the interior of the TatBC complex upon de-energetization of the membrane. This fully agrees with fluorescence-studies that showed TatA release upon addition of uncoupler that cause dissipation of the pmf 79. If TatB has to exchange with TatA for a successful TatA ring-opening and -lifting at the lateral opening, the TatA that is bridging the TatBC complex dissociates when the TatC TM5/TM6 domain moves back, and TatB reassociates with the TatC TM5/TM6 domain. After sealing of the membrane, the ring thus floats on the membrane surface, as supported by fluorescence-tracking experiments 56. Dissociated rings likely spontaneously close, and the transported but still membrane-anchored substrate can become captured in closed rings. Since substrate-binding had supported ring formation and a conformational transition of the TatA amphipathic helices that exposed the hinge to the cytoplasm, the lack of substrate contacts on the *cis* side after transport reverses the conformational transition and thus destabilizes the ring, which permits signal peptide release and post-translocation maturation processes (such as signal peptide cleavage or protein complex formation), as well as the spontaneous reinsertion of the TatA transmembrane helix into the membrane, reaching again a low-energy state. Thereby, smaller assemblies of membrane-inserted TatA are generated that are ready to assemble to rings again after substrate-binding. TatBC independent substrate interactions may thus be important for targeting. Signal peptides can interact with lipid bilayers, and, depending on the substrate, signal peptides can be efficiently recruited from a lipid-inserted state 37, 80. Therefore, it is likely that this substrate-binding is facilitated by signal peptide insertion into the membrane. This would explain why TatA-substrate interactions were twin-arginine independent but depend on the signal peptide, although the mature domain contacts are also important 39. With *in vitro* systems that used highly overproduced TatABC components and radiolabeled Tat substrates, a TatA interaction was found to be twin-arginine- and even pmf-dependent, indicating that this *in vitro* system did not monitor processes that occurred prior to TatBC binding and signal peptide insertion 41. Note that although the TatBC-independent TatA interactions likely can target substrates to TatBC in a TatA ring that is already primed for translocation, it is not necessarily the same ring that is used for transport, as TatA-dependent targeting may not be the only path to TatBC. In principle, TatA rings need to be recruited to the translocation site in the course of targeting or they can assemble *de novo* at the translocon. In the latter case, the Tat substrate that is already positioned on the membrane at the lateral gate may support this process, but signal peptides alone already result in the formation of the required ordered oligomer at the TatBC complex 17. The dimensions of the ring imply a contact to more than only TatC TM5. Accordingly, a pmf-dependent predicted surface-exposed TatA contact site was discovered at TM4 of TatC in plant systems (pea plastids, L231), which is nearby 30. The pmf thus contributes to TatA binding, and also membrane-thinning at TatBC likely contributes to the recruitment of TatA oligomers, as a hydrophobic mismatch is generated by membrane-integral TatA 27, 57. Based on the observation that TatC can organize the alignment of multiple TatA tubes 62, we think that multiple rings can be associated with each TatBC complex, and possibly several TatBC complexes can be clustered together with multiple TatA rings. Such lateral binding sites are likely responsible for the residual TatA detected in purified TatBC complexes by Deme et al. 9. There is clear evidence from studies that did not involve fluorescent protein tags that TatBC complexes interact with large TatA assemblies 32, and that both TatA clusters as well as TatBC complexes are predominantly found near cell poles, supporting the view that the larger TatA assemblies cluster in close vicinity of TatBC 27, 62.

The release of the Tat substrate from TatBC sterically permits the movement of the TatC TM5/6 domain together with its bound TatB back into the resting position. As the initial movement of the domain depended on the membrane energetization, the movement back to the resting position is likely facilitated by the local depolarization of the membrane. As a consequence, it can be concluded that the free flow of protons and other ions through the membrane likely supports the recovery of the complex and thus is a requirement for Tat transport.

In this context, the highly controversial aspect of soluble TatA should be mentioned. The loosely membrane-attached TatA rings that are generated by the translocation mechanism could in principle also dissociate from the membrane and be able to attach again for reinsertion. This would explain the detection of soluble TatA in several organisms, such as *Bacillus subtilis* 81, 82, *Streptomyces lividans* 83, archaea 84, or plants 85, 86. In case of *Bacillus subtilis*, the soluble PhoD-specific TatA has been reported to play a role in substrate-targeting to the membrane 81, 82. Such a targeting function was proposed for *Streptomyces* as well 83. In *Streptomyces,* TatA and TatB have more overlapping functions and thus the systems more resemble TatAC systems of *Bacillus* 83. TatA and TatB are both also present in the cytoplasm, and part of the TatA and TatB can be washed from membranes by carbonate washes, which is why *Streptomyces* “TatB” could be rather a bifunctional TatA 87. These aspects were widely neglected in the Tat literature and should be reassessed.

### The Tat system requires a functional asymmetry

The above-described translocation mechanism involves changes of the membrane energetization at the translocation site that must be coordinated. While the twin-arginine recognition and hence substrate-binding functions at all three TatC subunits even in the absence of membrane energetization, the signal peptide insertase activity and the TatA ring extraction transition require a membrane energetization whereas membrane disruption depolarizes the translocation site. It is thus crucial for the Tat system to assure a sequential translocation, coordinated by TatBC complexes. There are in principle two alternative ways to achieve a functional asymmetry: (i) When translocation is initiated at one site, a *regulatory mechanism* can block the initiation of translocation at the two other sites, or (ii) a *structural asymmetry* can exist that permits translocation at only one site. TatAC systems of the *Bacillus*-type can only use the regulatory mechanism, which likely is functioning also in *E. coli* TatB ${}_{3}$ C ${}_{3}$ complexes. However, in phylogenetically distant systems, structural asymmetry may have evolved, as it is possible that TatC-containing complexes differ in the number of involved subunits. A good example may be the Tat system of the halophilic archaeon *Halobacterium salinarium*, which possesses in addition to a “normal” TatC1 a large TatC2, which consists of two fused TatC that are connected by two additional transmembrane helices and a large cytoplasmic loop 88. TatC1 and TatC2 are both encoded in direct vicinity, and together they likely form an asymmetric complex with only one functional substrate binding site.

## CONCLUSIONS

**Figure 5:**
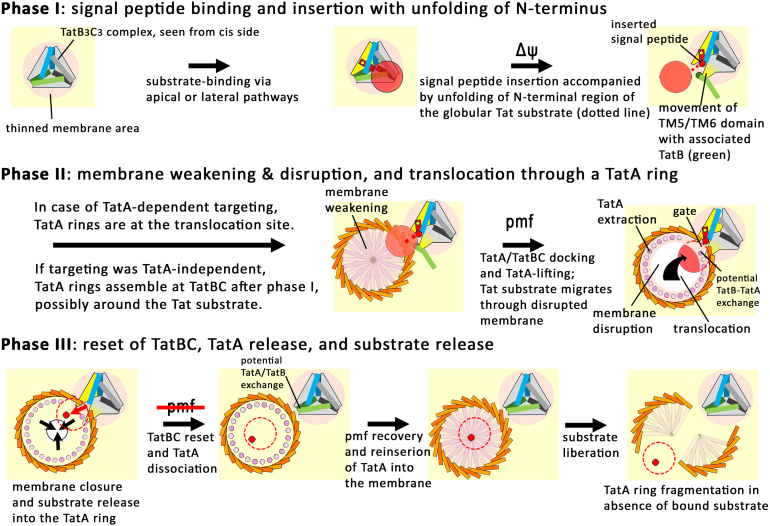
Schematic summary of the series of events that have to take place for Tat transport of folded proteins across membranes. The process can be divided in three phases that comprise several steps, as indicated. All stages show views from the cis side on the membrane. Light gray, TatC without bound substrate; yellow, TatC with bound substrate; blue, TatB that covers with its APH the bound signal peptide; green, TatB that blocks signal peptide insertion; red, substrate; dark red, signal peptide; yellow dot in signal peptide, RR-motif; light and dark orange, neighboring TatA APHs; light and dark pink, TatA transmembrane helices. When membrane-inserted, these helices are covered by a transparent yellow; when membrane-extracted, the helices are drawn as circles aligned at the inner side of the ring of amphipathic helices. Dashed red circle, globular substrate domain on the trans-side of the membrane ( = behind the membrane); dashed red line that connects the signal peptide with the globular domain, unfolded N-terminal region of the mature domain after signal peptide insertion.

After more than 30 years of Tat research, the mechanism was still unclear and diverse puzzling results have been obtained that were not well decoded. To propose a convincing mechanism for Tat transport, it was important to respect the technically sound unexplained findings as facts, resulting in the key questions highlighted in this review. The solution of the TatBC complex structure with bound Tat substrates and the AF3 prediction of a meaningful TatA ring were the last puzzle pieces, and now everything could be integrated into a model that can explain the mechanism on a molecular level. However, although the key predictions of our model are already supported by experimental evidence, more studies will be required to fully establish the principles postulated in this review. As schematically summarized in Figure 5, the transport occurs in two membrane energetization-dependent phases, with the signal peptide insertion and the membrane-disruption as main events, followed by a third phase for substrate release and translocon reset. Our model now permits to specifically address many details of the pathway that are still unclear, such as the questions: What determines the targeting pathway to the translocon? Are distinct pathways preferred by distinct substrates or organisms? Is there a structural and/or functional heterogeneity of Tat systems from phylogenetically distant groups of organisms? Does the exposed TatB (or bifunctional TatA) need to be exchanged at TatC-TM5 by a TatA of a TatA ring? Does the exposed TatB or the TatA/B binding site at TatC TM5 open existing TatA rings or assemble TatA rings newly at the translocation site? Do the observed diverse TatA ring sizes represent detergent-induced fragments of a single physiologically relevant ring, or can TatA rings adapt to the size of the translocated substrate? Although many findings are now understood, Tat research remains as exciting as it was at its very beginning more than 30 years ago.

## SUPPLEMENTAL MATERIAL

All supplemental data for this article are available online at http://microbialcell.com/researcharticles/2026a-brüser-microbial-cell/.

.

.

## CONFLICT OF INTEREST

The authors declare no conflict of interest.

## ABBREVIATIONS

AF3 – AlphaFold 3

APH – amphipathic helix

BN PAGE – blue native polyacrylamide gel electrophoresis

CCCP – carbonyl cyanide m-chlorophenyl hydrazone

CryoEM – cryo electron microscopy

ipTM – interface predicted template modelling

pTM – predicted template modelling

RR – twin-arginine

Tat – twin-arginine translocation

TM – transmembrane helix
