# Supplementary material for: The mechanism of Tat-dependent protein translocation
Source: Microb Cell. 2026 May 15;13:169–85. doi: 10.15698/mic2026.05.875 (PMC13186574; doi:10.15698/mic2026.05.875)
Supplement: Supplementary file 1 — . [file mic-13-169-s01.pdf]

Species and sequence ID and length	ipTM/pTM	top-view<br>AF3 pLDDT color code	APH assembly in the ring
Aquifex spec.<br>NPA41294.1 - 59 residues<br>Aquificota<br>extreme thermophilic bacterium	0.54/0.56	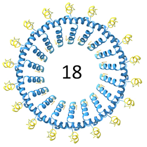	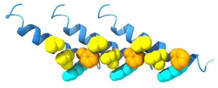
Desulfovibrio vulgaris<br>AAS96922.1 - 68 residues<br>Thermodesulfobacteriota<br>mesophilic bacterium	0.44/0.45	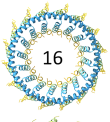	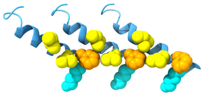
Methanococcus maripaludis<br>WP_197538631.1 - 70 residues<br>Methanobacteriota<br>mesophilic archaeon	0.42/0.44<br>(0.58/0.6)	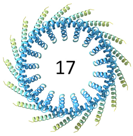	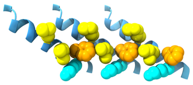
Thermoplasma volcanium<br>WP_010916629.1 - 100 residues<br>Thermoplasmatota<br>extreme thermophilic archaeon	0.40/0.42	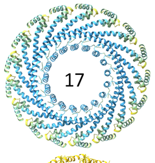	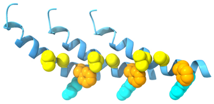
Frankia alni<br>WP_011604029.1 - 90 residues<br>Actinomycetota<br>mesophilic bacterium	0.39/0.41<br>(0.42/0.45)	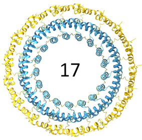	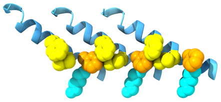
Bacillus subtilis TatAd<br>QGU25986.1 - 70 residues<br>Bacillota<br>mesophilic bacterium	0.39/0.41	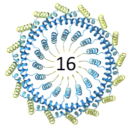	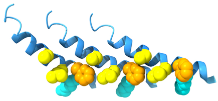
Escherichia coli TatE<br>NP_415160.1 - 67 residues<br>Pseudomonadota<br>mesophilic bacterium	0.23/0.25	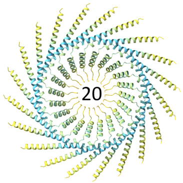	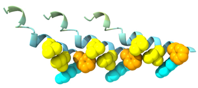
Chloroflexus aurantiacus<br>WP_448336613.1 - 62 residues<br>Chloroflexota<br>thermophilic bacterium	0.2/0.23	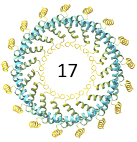	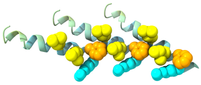
Bacillus subtilis TatAy<br>QGU25671.1 - 57 residues<br>Bacillota<br>mesophilic bacterium	0.19/0.22	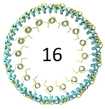	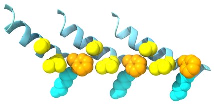
Escherichia coli TatA<br>NP_418280.4 - 89 residues<br>Pseudomonadota<br>mesophilic bacterium	0.19/0.20<br>20-24mers with same scores	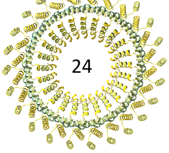	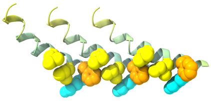

Figure S1: AlphaFold 3 predicts the same basic ring structure for TatA from diverse archaeal and bacterial phyla. Indicated are species names, specific TatA names (in case there are several in the species), GenBank accession numbers, sequence length, phylum and characteristics, the ipTM and pTM, and the number of protomers that form the shown best scoring ring. The list is ordered from highest scores (top) to lowest (bottom). The ring is colored by the AF3 pLDDT score color code. Note that the amphipathic helices always form the ring, even in case of the lowest scoring rings, whereas the N-terminal transmembrane helices are likely flexible, as are the C-terminal domains that strongly vary in sequence. Note that AF3 predicts highly variable C-terminal domain structures also the same protein and the C-terminus may be unstructured in many cases, as known for *Bacillus* TatAd (Lange et al. 2007), which is why only the ring of amphipathic helices and the inside located N-terminal helices are reliable, especially in conjunction with the known experimental evidence. In some cases, a truncation of the unstructured C-terminus can increase the ipTM and pTM scores (examples are indicated in brackets). The shown amphipathic helices with their FK motifs (orange, cyan) and interacting hydrophobic residues that trigger staggered ring formation (yellow) visualize the high degree of structural conservation of the ring in archaea and bacteria. Note that the conserved K is substituted by an R in *Chloroflexus*.

Supplemental Video S1: Likely movement of the TatC TM5/TM6 domain together with TatB, and signal peptide insertion at TatC. The video shows the movement and highlights the carboxyl group at TatC-D211 that is required for the movement in the electric field. Also, the three arginines are shown that may contribute to the movement. Note that a straight helix can be formed when the signal peptide fully extends into the opening generated by the movement. The video is based on PDB9E01 [9] and was created using ChimeraX 1.10.1 [88].
